# Fast Bayesian parameter estimation for stochastic logistic growth models

**DOI:** 10.1016/j.biosystems.2014.05.002

**Published:** 2014-08

**Authors:** Jonathan Heydari, Conor Lawless, David A. Lydall, Darren J. Wilkinson

**Affiliations:** Newcastle University, UK

**Keywords:** Kalman filter, Linear noise approximation, Logistic, Population growth, Stochastic modelling

## Abstract

The transition density of a stochastic, logistic population growth model with multiplicative intrinsic noise is analytically intractable. Inferring model parameter values by fitting such stochastic differential equation (SDE) models to data therefore requires relatively slow numerical simulation. Where such simulation is prohibitively slow, an alternative is to use model approximations which do have an analytically tractable transition density, enabling fast inference. We introduce two such approximations, with either multiplicative or additive intrinsic noise, each derived from the linear noise approximation (LNA) of a logistic growth SDE. After Bayesian inference we find that our fast LNA models, using Kalman filter recursion for computation of marginal likelihoods, give similar posterior distributions to slow, arbitrarily exact models. We also demonstrate that simulations from our LNA models better describe the characteristics of the stochastic logistic growth models than a related approach. Finally, we demonstrate that our LNA model with additive intrinsic noise and measurement error best describes an example set of longitudinal observations of microbial population size taken from a typical, genome-wide screening experiment.

## Graphical abstract

**Figure:**
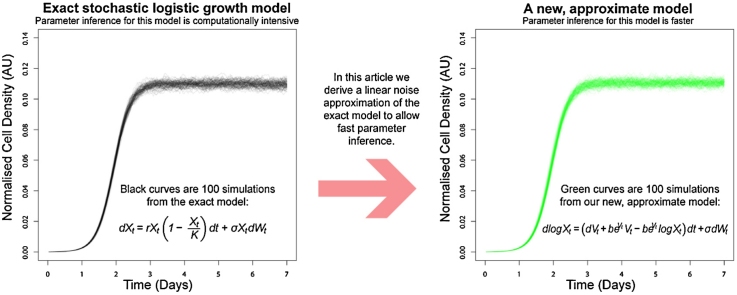

## 1 Introduction

Stochastic models simultaneously describe dynamics and noise or heterogeneity in real systems (Chen et al., 2010). For example, stochastic models are increasingly recognised as necessary tools for understanding the behaviour of complex biological systems (Wilkinson, 2011, Wilkinson, 2009) and are also used to capture uncertainty in financial market behaviour (Kijima, 2013, Koller, 2012). Many such models are written as continuous stochastic differential equations (SDEs) which often do not have analytical solutions and are slow to evaluate numerically compared to their deterministic counterparts. Simulation speed is often a particularly critical issue when inferring model parameter values by comparing simulated output with observed data (Hurn et al., 2007).

For SDE models where no explicit expression for the transition density is available, it is possible to infer parameter values by simulating a latent process using a data augmentation approach (Golightly and Wilkinson, 2005). However, this method is computationally intensive and not practical for all applications. When fast inference for SDEs is important, for example for real-time analysis as part of decision support systems or for big data inference problems where we simultaneously fit models to many thousands of datasets (e.g. Heydari et al. (2012)), we need an alternative approach. Here we demonstrate one such approach: developing an analytically tractable approximation to the original SDE, by making linear noise approximations (LNAs) (Kurtz, 1970, Kurtz, 1971, Van Kampen, 2011). We apply this approach to a SDE describing logistic population growth for the first time.

The logistic model of population growth, an ordinary differential equation (ODE) describing the self-limiting growth of a population of size *x*_*t*_ at time *t*, was developed by Verhulst (1845)

(1) $$\frac{{\mathrm{dx}}_{t}}{\mathrm{dt}}={\mathrm{rx}}_{t}\left(1-\frac{x_{t}}{K}\right).$$

The ODE has the following analytic solution:

(2) $$x_{t}=\frac{K}{1+{\mathrm{Qe}}^{-\mathrm{rt}}},$$

(3) $$\mathrm{where}\;Q=\left(\frac{K}{P}-1\right)e^{{\mathrm{rt}}_{0}},\;P=x_{t_{0}}\;\mathrm{and}\;t\geq t_{0}.$$

The model describes a population growing from an initial size *P* with an intrinsic growth rate *r*, undergoing approximately exponential growth which slows as the availability of some critical resource (e.g. nutrients or space) becomes limiting (Peleg et al., 2007). Ultimately, population density saturates at the carrying capacity (maximum achievable population density) *K*, once the critical resource is exhausted. Where further flexibility is required, generalized forms of the logistic growth process (Tsoularis and Wallace, 2002) may be used instead.

To account for uncertainty about processes affecting population growth which are not explicitly described by the deterministic logistic model, we can include a term describing intrinsic noise and consider a SDE version of the model. By adding a term representing multiplicative intrinsic noise to the ODE in (1) we arrive at a model, first introduced by Capocelli and Ricciardi (1974), which we refer to as the stochastic logistic growth model (SLGM):

(4) $${\mathrm{dX}}_{t}={\mathrm{rX}}_{t}\left(1-\frac{X_{t}}{K}\right)\mathrm{dt}+\sigma X_{t}{\mathrm{dW}}_{t},$$

where $P=x_{t_{0}}$ and is independent of Wiener process *W*_*t*_, *t* ≥ *t*_0_.

The Kolmogorov forward equation has not been solved for (4), therefore no explicit expression for the transition density is available.

Alternative stochastic formulations of the logistic ODE can be generated (Campillo et al., 2013).

While not equivalent to (4), SDEs derived from logistic growth Markov jump processes (MJPs) (Feller, 1939) are available within the literature (Ross et al., 2006, Ross et al., 2009). The intrinsic noise in MJPs tends to zero with larger population sizes, while (4) introduces an additional parameter *σ* that allows us to tune the amount of noise in the system that is not directly associated with the noise due to the discreteness of the process (demographic noise). For the yeast populations that we model during the analysis of high-throughput screens (see Section 5.2) we observe fluctuations much larger than those consistent with demographic noise, especially in the stationary phase. Consequently SDEs derived from MJPs do not adequately describe our data. Therefore, we find that the SLGM in (4) is the more appropriate model for estimating logistic growth parameters of large populations.

Román-Román and Torres-Ruiz (2012) derive a diffusion process (which we label RRTR) from a reparameterisation of the logistic growth model. We use the RRTR as an approximation of the SLGM. Unlike the SLGM, the RRTR has a transition density that can be derived explicitly, enabling fast inference. The Bayesian approach can be applied in a natural way to carry out parameter inference for state space models with tractable transition densities (West and Harrison, 1997). The transition density is used to describe the Markovian evolution of the state process *S*_*t*_. A state space model also describes the probabilistic dependence between an observation process variable *X*_*t*_ and state process *S*_*t*_ via a measurement error model.

The Kalman filter (Kalman, 1960) is typically used to infer a hidden state process of interest *S*_*t*_ and is an optimal estimator, minimising the mean square error of estimated parameters when all noise in the system can be assumed to be Gaussian. The main assumptions of the Kalman filter are that the underlying system is a linear dynamical system and that the noise has known first and second moments. Here, we use the Kalman filter in a different way: to reduce computational time in a parameter inference algorithm by recursively computing the marginal likelihood (West and Harrison, 1997).

The RRTR can be fit to data within an acceptable time frame by assuming multiplicative measurement error to give a linear Gaussian structure, allowing us to use a Kalman filter for inference.

We introduce two new first order linear noise approximations (LNAs) (Wallace, 2010, Komorowski et al., 2009) of (4), one with multiplicative and one with additive intrinsic noise, which we label LNAM and LNAA respectively. The LNA reduces a SDE to a linear SDE with additive noise, which can be solved to give an explicit expression for the transition density. The LNA assumes the solution of a diffusion process *Y*_*t*_ can be written as $Y_{t}=v_{t}+Z_{t}$ (a deterministic part $v_{t}$ and stochastic part *Z*_*t*_), where *Z*_*t*_ remains small for all $t\in {\mathbb{R}}_{\geq 0}$. The LNA is useful when a tractable solution to a SDE cannot be found. Typically the LNA is used to reduce an SDE to a Ornstein-Uhlenbeck process which can be solved explicitly. Ornstein–Uhlenbeck processes are linear Gaussian, so time-discretising the resulting LNA will therefore give a linear Gaussian state space model with an analytically tractable transition density. We derive transition densities for the two approximate models and construct a Kalman filter by choosing measurement noise to be either multiplicative or additive to retain linear Gaussian structure. Exact simulations from the SGLM are compared with each of the three approximate models. We compare the utility of each of the approximate models for parameter inference by comparing simulations with both synthetic and real datasets.

## 2 The Román-Román and Torres-Ruiz (2012) diffusion process

Román-Román and Torres-Ruiz (2012) present a logistic growth diffusion process (RRTR) which has a transition density that can be written explicitly, allowing inference of model parameter values from discrete sampling trajectories.

The RRTR is derived from the following ODE:

(5) $$\frac{{\mathrm{dx}}_{t}}{\mathrm{dt}}=\frac{\mathrm{Qr}}{e^{\mathrm{rt}}+Q}x_{t}.$$

The solution to (5) is given in (2) (it has the same solution as (1)). Román-Román and Torres-Ruiz (2012) derive the RRTR from a reparameterisation of the logistic growth model (2) for which the limit value depends on the initial one. Using the reparameterisation introduced by Román-Román and Torres-Ruiz (2012) it is also possible to carry out inference in situations where there are several observed trajectories of the same phenomenon, each of them showing logistic growth but with a different initial value.

Román-Román and Torres-Ruiz (2012) see (5) as a generalisation of the Malthusian growth model with a deterministic, time-dependent fertility *h*(*t*) = *Qr*/(*e*^*rt*^ + *Q*), and replace this with *Qr*/(*e*^*rt*^ + *Q*) + *σW*_*t*_ to obtain the following approximation to the SLGM:

(6) $${\mathrm{dX}}_{t}=\frac{\mathrm{Qr}}{e^{rt}+Q}X_{t}dt+\sigma X_{t}{\mathrm{dW}}_{t},$$

where *Q* and *P* are defined by (3). The process described in (6) is a particular case of the lognormal process with exogenous factors, therefore an exact transition density is available (Gutiérrez et al., 2006). The transition density for *Y*_*t*_, where *Y*_*t*_ = log(*X*_*t*_), can be written:

(7) $$\begin{array}{cc} (Y_{t_{i}}|Y_{t_{i-1}} & =y_{t_{i-1}})∼\text{N}\left(\mu_{t_{i}},\Xi_{t_{i}}\right),\\ \mathrm{where} & a=r,\;b=\frac{r}{K},\\ \mu_{t_{i}}= & \log(y_{t_{i-1}})+\log\left(\frac{1+{\mathrm{be}}^{-{\mathrm{at}}_{i}}}{1+{\mathrm{be}}^{-{\mathrm{at}}_{i-1}}}\right)-\frac{\sigma^{2}}{2}(t_{i}-t_{i-1})\;\mathrm{and}\\ \Xi_{t_{i}} & =\sigma^{2}(t_{i}-t_{i-1}). \end{array}$$

We chose a lognormal (multiplicative) measurement error model in order to construct a linear Gaussian structure, enabling fast inference through the use of a Kalman filter for marginal likelihood computation.

## 3 Linear noise approximation with multiplicative noise

We now take a different approach to approximating the SLGM (4), which will turn out to be closer to the exact solution of the SLGM than the RRTR (6). Starting from the original model (4), we apply Itô's lemma with the transformation *f*(*X*_*t*_) ≡ *Y*_*t*_ = log *X*_*t*_ to obtain the following Itô drift-diffusion process:

(8) $${\mathrm{dY}}_{t}=\left(r-\frac{1}{2}\sigma^{2}-\frac{r}{K}e^{Y_{t}}\right)\mathrm{dt}+\sigma {\mathrm{dW}}_{t}.$$

The log transformation from multiplicative to additive noise gives a constant diffusion term which will allow the LNA to give a better approximation of the diffusion term than it could on the original scale.

The LNA can be viewed as a first order Taylor expansion of an approximating SDE about a deterministic solution (Fearnhead et al., 2014). We now separate the process *Y*_*t*_ into a deterministic part $v_{t}$ and a stochastic part *Z*_*t*_ so that $Y_{t}=v_{t}+Z_{t}$ and consequently *dY*_*t*_ = *dv*_*t*_ + *dZ*_*t*_. We choose $v_{t}$ to be the solution of the deterministic part

(9) $${\mathrm{dv}}_{t}=\left(r-\frac{1}{2}\sigma^{2}-\frac{r}{K}e^{v_{t}}\right)\mathrm{dt}.$$

Without loss of generality we set *t*_0_ = 0. After redefining our notation as follows: $a=r-\frac{\sigma^{2}}{2}$ and $b=\frac{r}{K}$, we solve (9) for $v_{t}$,

(10) $$v_{t}=\log\left(\frac{{\mathrm{aPe}}^{\mathrm{at}}}{\mathrm{bP}(e^{\mathrm{at}}-1)+a}\right).$$

We now write down an expression for *dZ*_*t*_, where *dZ*_*t*_ = *dY*_*t*_ − *dv*_*t*_:

$${\mathrm{dZ}}_{t}=\left(a-{\mathrm{be}}^{Y_{t}}\right)\mathrm{dt}+\sigma {\mathrm{dW}}_{t}-\left(a-{\mathrm{be}}^{v_{t}}\right)\mathrm{dt}$$

We then substitute in $Y_{t}=v_{t}+Z_{t}$ and simplify the expression to give

$${\mathrm{dZ}}_{t}=b\left(e^{v_{t}}-e^{v_{t}+Z_{t}}\right)\mathrm{dt}+\sigma {\mathrm{dW}}_{t}.$$

*dZ*_*t*_ is a non-linear SDE that cannot be solved explicitly. We use the LNA to obtain a SDE that can be solved: we make a first-order approximation of *e*^*Z*_*t*_^ ≈ 1 + *Z*_*t*_ and then simplify to give

(11) $${\mathrm{dZ}}_{t}=-{\mathrm{be}}^{v_{t}}Z_{t}\mathrm{dt}+\sigma {\mathrm{dW}}_{t}.$$

This process is a particular case of the time-varying Ornstein–Uhlenbeck process, which can be solved explicitly. The transition density for *Y*_*t*_ (derivation in Appendix A) is then:

(12) $$\begin{array}{cc} (Y_{t_{i}}|Y_{t_{i-1}} & =y_{t_{i-1}})∼\text{N}(\mu_{t_{i}},\Xi_{t_{i}}),\\ \mathrm{redefine}\;\;y_{t_{i-1}} & =v_{t_{i-1}}+z_{t_{i-1}},Q=\left(\frac{a/b}{P}-1\right),\\ \mu_{t_{i}} & =y_{t_{i-1}}+\log\left(\frac{1+{\mathrm{Qe}}^{-{\mathrm{at}}_{i-1}}}{1+{\mathrm{Qe}}^{-{\mathrm{at}}_{i}}}\right)+e^{-a(t_{i}-t_{i-1})}\frac{1+{\mathrm{Qe}}^{-{\mathrm{at}}_{i-1}}}{1+{\mathrm{Qe}}^{-{\mathrm{at}}_{i}}}z_{t_{i-1}}\;\mathrm{and}\\ \Xi_{t_{i}} & =\sigma^{2}\left[\frac{4Q(e^{{\mathrm{at}}_{i}}-e^{{\mathrm{at}}_{i-1}})+e^{2{\mathrm{at}}_{i}}-e^{2{\mathrm{at}}_{i-1}}+2{\mathrm{aQ}}^{2}(t_{i}-t_{i-1})}{2a{\left(Q+e^{{\mathrm{at}}_{i}}\right)}^{2}}\right]. \end{array}$$

The LNA of the SLGM with multiplicative intrinsic noise (LNAM) can then be written as

$${\mathrm{dY}}_{t}=\left({\mathrm{dv}}_{t}+{\mathrm{be}}^{v_{t}}v_{t}-{\mathrm{be}}^{v_{t}}Y_{t}\right)\mathrm{dt}+\sigma {\mathrm{dW}}_{t},$$

or alternatively in terms of *X*_*t*_,

$${\mathrm{dX}}_{t}=\left[X_{t}\left({\mathrm{dv}}_{t}+{\mathrm{be}}^{v_{t}}v_{t}-{\mathrm{be}}^{v_{t}}\log X_{t}+\frac{1}{2}\sigma^{2}\right)\right]\mathrm{dt}+\sigma X_{t}{\mathrm{dW}}_{t},$$

where *Y*_*t*_ = log *X*_*t*_, *X*_0_ = *P* is independent of *W*_*t*_ and *t* ≥ 0.

Similar to the RRTR, we chose a lognormal (multiplicative) measurement error model in order to construct a linear Gaussian structure, enabling fast inference through the use of a Kalman filter for marginal likelihood computation.

Note that the RRTR given in (6) can be similarly derived using a zero-order noise approximation (*e*^*Z*_*t*_^ ≈ 1) instead of the LNA.

## 4 Linear noise approximation with additive noise

As in Section 3, we start from the SLGM, given in (4). Without first log transforming the process, the LNA will lead to a worse approximation to the diffusion term of the SLGM, but we will see in the coming sections that there are nevertheless advantages. We separate the process *X*_*t*_ into a deterministic part $v_{t}$ and a stochastic part *Z*_*t*_ so that *dX*_*t*_ = *dv*_*t*_ + *dZ*_*t*_ and consequently $X_{t}=v_{t}+Z_{t}$. We choose $v_{t}$ to be the solution of the deterministic part

$${\mathrm{dv}}_{t}=\left({\mathrm{rv}}_{t}-\frac{r}{K}v_{t}^{2}\right)\mathrm{dt}.$$

Without loss of generality we set *t*_0_ = 0. After redefining our previous notation as follows: *a* = *r* and $b=\frac{r}{K}$, we solve *dv*_*t*_ to give:

(13) $$v_{t}=\frac{{\mathrm{aPe}}^{\mathrm{at}}}{\mathrm{bP}(e^{\mathrm{at}}-1)+a}.$$

We now solve *dZ*_*t*_, where *dZ*_*t*_ = *dX*_*t*_ − *dv*_*t*_. Expressions for both *dX*_*t*_ and *dv*_*t*_ are known:

$${\mathrm{dZ}}_{t}=({\mathrm{aX}}_{t}-{\mathrm{bX}}_{t}^{2})\mathrm{dt}+\sigma X_{t}{\mathrm{dW}}_{t}-({\mathrm{av}}_{t}-{\mathrm{bv}}_{t}^{2})\mathrm{dt}.$$

We then substitute in $X_{t}=v_{t}+Z_{t}$ and simplify the expression to give

$${\mathrm{dZ}}_{t}=(a-2{\mathrm{bv}}_{t})Z_{t}-{\mathrm{bZ}}_{t}^{2}\mathrm{dt}+\left(\sigma v_{t}+\sigma Z_{t}\right){\mathrm{dW}}_{t}.$$

*dZ*_*t*_ is a non-linear SDE that cannot be solved explicitly. We use the LNA to obtain a SDE that can be solved: we set second order term $-{\mathrm{bZ}}_{t}^{2}\mathrm{dt}=0$ and *σZ*_*t*_*dW*_*t*_ = 0 such that the following SDE is linear in the narrow sense (Kloeden and Platen, 1992) (additive noise) to give

(14) $${\mathrm{dZ}}_{t}=(a-2{\mathrm{bv}}_{t})Z_{t}\mathrm{dt}+\sigma v_{t}{\mathrm{dW}}_{t}.$$

This approximate process is a particular case of the Ornstein–Uhlenbeck process, which can be solved. The transition density for *X*_*t*_ (derivation in Appendix B) is then

(15) $$\begin{array}{cc} (X_{t_{i}}|X_{t_{i-1}} & =x_{t_{i-1}})∼N(\mu_{t_{i}},\Xi_{t_{i}}),\\ \mathrm{where}\;\;x_{t_{i-1}} & =v_{t_{i-1}}+z_{t_{i-1}},\\ \mu_{t_{i}} & =x_{t_{i-1}}+\left(\frac{{\mathrm{aPe}}^{{\mathrm{at}}_{i}}}{\mathrm{bP}(e^{{\mathrm{at}}_{i}}-1)+a}\right)-\left(\frac{{\mathrm{aPe}}^{{\mathrm{at}}_{i-1}}}{\mathrm{bP}(e^{{\mathrm{at}}_{i-1}}-1)+a}\right)+e^{a(t_{i}-t_{i-1})}{\left(\frac{\mathrm{bP}(e^{{\mathrm{at}}_{i-1}}-1)+a}{\mathrm{bP}(e^{{\mathrm{at}}_{i}}-1)+a}\right)}^{2}Z_{t_{i-1}}\\ \mathrm{and} & \\ \Xi_{t} & =\frac{1}{2}\sigma^{2}{\mathrm{aP}}^{2}e^{2{\mathrm{at}}_{i}}{\left(\frac{1}{\mathrm{bP}(e^{{\mathrm{at}}_{i}}-1)+a}\right)}^{4}\times [b^{2}P^{2}(e^{2{\mathrm{at}}_{i}}-e^{2{\mathrm{at}}_{i-1}})+4\mathrm{bP}(a-\mathrm{bP})(e^{{\mathrm{at}}_{i}}-e^{{\mathrm{at}}_{i-1}})+2a(t_{i}-t_{i-1}){\left(a-\mathrm{bP}\right)}^{2}]. \end{array}$$

The LNA of the SLGM, with additive intrinsic noise (LNAA) can then be written as

$$\begin{array}{cc} {\mathrm{dX}}_{t}=\left[b{v_{t}}^{2}+\left(a-2{\mathrm{bv}}_{t}\right)X_{t}\right]\mathrm{dt}+\sigma v_{t}{\mathrm{dW}}_{t}, & \end{array}$$

where *X*_0_ = *P* independent of *W*_*t*_ and *t* ≥ 0. We chose a normal (additive) measurement error model in order to construct a linear Gaussian structure, enabling fast inference through the use of a Kalman filter for marginal likelihood computation.

## 5 Simulation and Bayesian inference for the stochastic logistic growth equation and approximations

**Fig. 1:**
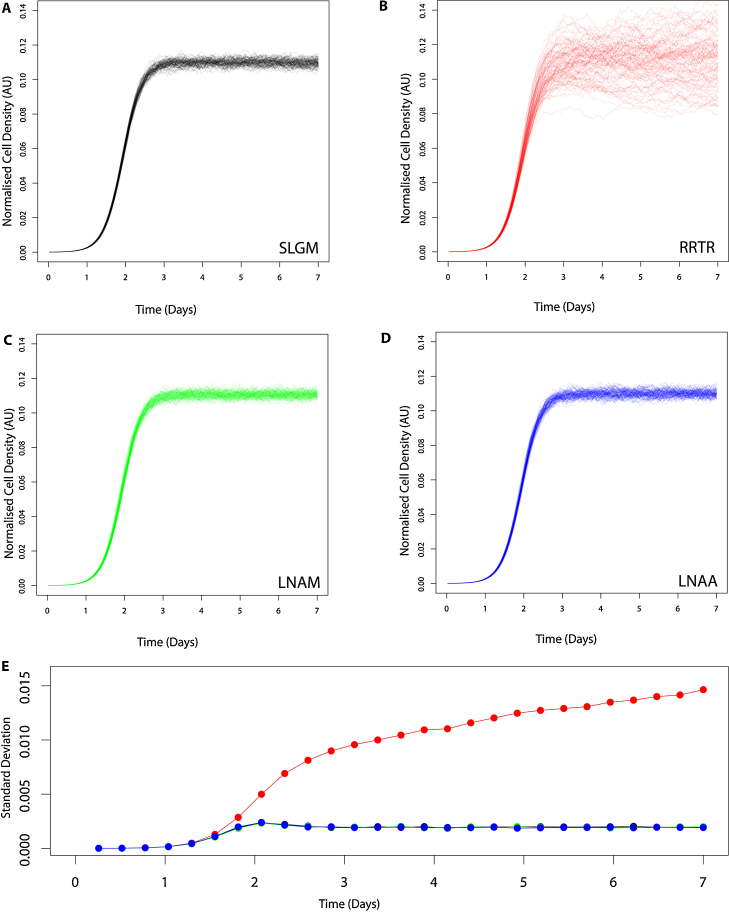
Forward trajectories (no. of simulations=100) for a logistic SDE and approximations. See Table 2 for parameter values. (A) The stochastic logistic growth model (SLGM). (B) The Román-Román and Torres-Ruiz (2012) (RRTR) approximation. (C) The linear noise approximation with multiplicative intrinsic noise (LNAM). (D) The linear noise approximation with additive intrinsic noise (LNAA). (E) Standard deviations of simulated trajectories over time for the SLGM (black), RRTR (red), LNAM (green) and LNAA (blue). (For interpretation of the references to color in this figure legend, the reader is referred to the web version of the article.)

To compare the accuracy of each of the three approximations for the SLGM, we first compare simulated forward trajectories from the RRTR, LNAM and LNAA with simulated forward trajectories from the SLGM (Fig. 1). We use the Euler-Maruyama (E-M) method (Kloeden and Platen, 1992) with very fine discretisation to give arbitrarily exact simulated trajectories from each SDE.

The LNAA and LNAM trajectories are visually indistinguishable from the SLGM (Fig. 1A–D). On the other hand, population sizes simulated with the RRTR display large deviations from the mean as the population approaches its stationary phase when compared to the SLGM (Fig. 1A and B). The RRTR, see (6), which can be derived from a zero-order noise approximation of the SLGM, lacks a mean reverting property found in the LNAM and LNAA. Fig. 1E further highlights the increases in variation as the population approaches stationary phase for simulated trajectories of the RRTR, in contrast to the SLGM and our LNA models.

To compare the approximate models across a large parameter space, in Table 1 we present mean squared errors (MSEs) for the mean growth and standard deviations, using forward simulations. Table 1 shows that, within the range of parameters considered, the LNAM and LNAA better approximate the mean curve of the SLGM than the RRTR, with ∼10 and ∼5 times lower MSE than the RRTR respectively. Similarly, Table 1 shows that the LNAM and LNAA better approximate the standard deviation of the SLGM than the RRTR, with ∼1000 and ∼1300 times less than the RRTR respectively. This observation is consistent with the RRTR lacking a mean reverting property. Both the LNAM and LNAA have good (low) MSE, with the LNAM slightly better overall, as expected.

**Table 1:** Mean squared errors (MSEs) between our approximate models and the SLGM are calculated for the mean growth of forward simulated trajectories over time (100 trajectories, each with 27 time points evenly spaced across *t* ∈ (0, 7)). MSEs are calculated across the following parameter space, *K* ∈ (0, 1), *r* ∈ (0, 8), *P* ∈ (0, 0.005) and *σ* ∈ (0, 0.6), evaluating at five evenly spaced values for each parameter range, giving a total of 5^4^ = 625 combinations. An average MSE is then calculated across the 625 MSEs obtained. Similarly an average MSE is calculated for the standard deviations of forward simulated trajectories over time, evaluating at each of the 27 time points.

Model	RRTR	LNAM	LNAA
Average MSE for mean growth	0.051	0.0043	0.010
Average MSE for standard deviation	2.024	0.0021	0.0016

### 5.1 Bayesian parameter inference with approximate models

To compare the quality of parameter inference using each of these approximations we simulated synthetic time-course data from the SLGM and combined this with either lognormal or normal measurement error. Carrying out Bayesian inference with broad priors (see (16), (17)) we compared posterior parameter estimates using each approximation with values used to generate the synthetic dataset. The synthetic time-course datasets consist of 27 time points generated using the E-M method with a fine discretisation (Kloeden and Platen, 1992). Parameter values used to simulate time courses, see Table 2, were chosen to cover the range observed in growth curves of healthy yeast cultures considered in the next section.

**Table 2:** Bayesian state space model parameter posterior means, standard deviations and true values for Fig. 2, Fig. 3 and 4. True values for the simulated data used for Fig. 1, Fig. 2, Fig. 3 are also given.

*Panel*	*Model*	$\hat{K}$		$\hat{r}$		$\hat{P}$		$\hat{\nu }$		$\hat{\sigma }$	
Fig. 2, *SLGM with lognormal error*											
A	SGLM+L	0.150	(0.001)	2.982	(0.014)	1.002 × 10^−04^	(1.112 × 10^−06^)	3.860 × 10^−03^	(2.127 × 10^−03^)	0.017	(0.005)
B	RRTR	0.150	(0.003)	2.990	(0.011)	9.931 × 10^−05^	(1.069 × 10^−06^)	5.684 × 10^−03^	(2.360 × 10^−03^)	0.012	(0.006)
C	LNAM	0.150	(0.001)	2.988	(0.013)	9.980 × 10^−05^	(1.124 × 10^−06^)	4.140 × 10^−03^	(2.180 × 10^−03^)	0.016	(0.005)
D	LNAA	0.150	(0.001)	3.005	(0.020)	9.647 × 10^−05^	(2.946 × 10^−06^)	3.099 × 10^−05^	(2.534 × 10^−05^)	0.019	(0.003)
E	SGLM+L	0.110	(0.001)	3.975	(0.047)	5.054 × 10^−05^	(1.568 × 10^−06^)	6.159 × 10^−03^	(5.527 × 10^−03^)	0.051	(0.014)
F	RRTR	0.109	(0.007)	3.984	(0.035)	5.046 × 10^−05^	(1.137 × 10^−06^)	5.928 × 10^−03^	(4.596 × 10^−03^)	0.037	(0.009)
G	LNAM	0.110	(0.001)	3.985	(0.046)	5.043 × 10^−05^	(1.580 × 10^−06^)	6.188 × 10^−03^	(5.191 × 10^−03^)	0.052	(0.013)
H	LNAA	0.110	(0.001)	3.959	(0.067)	5.207 × 10^−05^	(4.310 × 10^−06^)	4.540 × 10^−05^	(4.395 × 10^−05^)	0.059	(0.010)
I	SGLM+L	0.300	(0.001)	5.997	(0.029)	1.962 × 10^−05^	(4.041 × 10^−07^)	9.543 × 10^−03^	(4.035 × 10^−03^)	0.024	(0.015)
J	RRTR	0.301	(0.004)	6.015	(0.017)	1.943 × 10^−05^	(2.835 × 10^−07^)	1.241 × 10^−02^	(2.307 × 10^−03^)	0.008	(0.006)
K	LNAM	0.300	(0.001)	6.015	(0.031)	1.953 × 10^−05^	(4.202 × 10^−07^)	8.943 × 10^−03^	(4.252 × 10^−03^)	0.027	(0.016)
L	LNAA	0.300	(0.001)	6.037	(0.067)	1.895 × 10^−05^	(1.502 × 10^−06^)	8.122 × 10^−05^	(1.596 × 10^−04^)	0.047	(0.008)
Fig. 3, *SLGM with normal error*											
A	SLGM+N	0.150	(0.002)	3.099	(0.085)	9.299 × 10^−05^	(7.305 × 10^−06^)	5.326 × 10^−03^	(1.009 × 10^−03^)	0.059	(0.030)
B	RRTR	0.213	(0.123)	1.368	(0.263)	4.552 × 10^−03^	(2.118 × 10^−03^)	2.539 × 10^−01^	(1.097 × 10^−01^)	0.419	(0.129)
C	LNAM	0.171	(0.033)	1.580	(0.271)	5.241 × 10^−03^	(2.048 × 10^−03^)	2.054 × 10^−01^	(7.805 × 10^−02^)	0.473	(0.051)
D	LNAA	0.150	(0.002)	2.990	(0.262)	1.189 × 10^−04^	(7.099 × 10^−05^)	5.490 × 10^−03^	(1.060 × 10^−03^)	0.053	(0.033)
E	SLGM+N	0.109	(0.001)	4.183	(0.074)	4.390 × 10^−05^	(4.129 × 10^−06^)	9.679 × 10^−04^	(2.806 × 10^−04^)	0.057	(0.012)
F	RRTR	0.157	(0.087)	2.631	(0.337)	4.398 × 10^−04^	(1.678 × 10^−04^)	1.040 × 10^−01^	(1.009 × 10^−01^)	0.374	(0.162)
G	LNAM	0.116	(0.009)	3.019	(0.374)	4.967 × 10^−04^	(1.397 × 10^−04^)	3.346 × 10^−02^	(4.309 × 10^−02^)	0.475	(0.044)
H	LNAA	0.110	(0.001)	4.010	(0.158)	5.012 × 10^−05^	(1.443 × 10^−05^)	1.093 × 10^−03^	(3.638 × 10^−04^)	0.053	(0.013)
I	SLGM+N	0.305	(0.003)	5.267	(0.125)	3.263 × 10^−04^	(3.407 × 10^−05^)	1.119 × 10^−02^	(1.974 × 10^−03^)	0.045	(0.031)
J	RRTR	0.314	(0.057)	3.030	(0.233)	1.307 × 10^−03^	(2.897 × 10^−04^)	2.228 × 10^−01^	(3.708 × 10^−02^)	0.075	(0.086)
K	LNAM	0.313	(0.020)	3.392	(0.430)	1.118 × 10^−03^	(3.269 × 10^−04^)	1.176 × 10^−01^	(8.435 × 10^−02^)	0.360	(0.165)
L	LNAA	0.302	(0.002)	5.862	(0.523)	2.890 × 10^−05^	(2.599 × 10^−05^)	8.774 × 10^−03^	(1.466 × 10^−03^)	0.041	(0.028)
Fig. 4, *observed yeast data*											
A	SLGM+L	0.110	(0.007)	4.098	(0.299)	7.603 × 10^−06^	(3.206 × 10^−06^)	3.457 × 10^−01^	(5.319 × 10^−02^)	0.113	(0.109)
B	SLGM+N	0.110	(0.003)	3.905	(0.173)	1.044 × 10^−05^	(3.086 × 10^−06^)	1.852 × 10^−04^	(7.460 × 10^−05^)	0.167	(0.028)
C	RRTR	0.114	(0.026)	3.764	(0.201)	1.079 × 10^−05^	(3.155 × 10^−06^)	3.379 × 10^−01^	(4.840 × 10^−02^)	0.078	(0.077)
D	LNAM	0.110	(0.011)	3.777	(0.216)	1.077 × 10^−05^	(3.277 × 10^−06^)	3.362 × 10^−01^	(5.137 × 10^−02^)	0.104	(0.108)
E	LNAA	0.109	(0.003)	3.832	(0.198)	1.069 × 10^−05^	(3.680 × 10^−06^)	1.769 × 10^−04^	(6.607 × 10^−05^)	0.164	(0.033)

True values	*K*	*r*	*P*	*ν*	*σ*
Fig. 1, panels A–D	0.11	4	0.00005	N/A	0.05
Fig. 2 and 3, panels A–D	0.15	3	0.0001	0.005	0.01
Fig. 2 and 3, panels E–H	0.11	4	0.00005	0.001	0.05
Fig. 2 and 3, panels I–L	0.3	6	0.0002	0.01	0.02

We formulate our inference problem as a dynamic linear state space model. To allow fast parameter inference we have chosen our measurement error models to give linear Gaussian structures and construct a Kalman filter recursion for marginal likelihood computation (Appendix D). We therefore assume lognormal (multiplicative) error for the RRTR and LNAM, and for the LNAA we assume normal (additive) measurement error. Dependent variable $y_{t_{i}}$ and independent variable {*t*_*i*_, *i* = 1, …, *N*} are data input to the model (where *t*_*i*_ is the time at point *i* and *N* is the number of time points). *X*_*t*_ is the state process, describing the population size.

The state space model for the RRTR and LNAM is as follows:

(16) $$\begin{array}{cc} \log(y_{t_{i}}) & ∼\text{N}(X_{t_{i}},\nu^{2}),\\ \left(X_{t_{i}}|X_{t_{i-1}}=x_{t_{i-1}}\right) & ∼\text{N}(\mu_{t_{i}},\Xi_{t_{i}}),\;\mathrm{where}\;\;x_{t_{i}}=v_{t_{i}}+z_{t_{i}}, \end{array}$$

$\mu_{t_{i}}$ and $\Xi_{t_{i}}$ are given by (7), (12) for the RRTR and LNAM respectively. Priors are as follows:

$$\begin{array}{cccccc} \log X_{0}\equiv \log\;P & ∼\text{N}(\mu_{P},{\tau_{P}}^{-1}), & \;\;\log\;K & ∼\text{N}(\mu_{K},{\tau_{K}}^{-1}), & \;\;\log\;r & ∼\text{N}(\mu_{r},{\tau_{r}}^{-1}),\\ \log\;\nu^{-2} & ∼\text{N}(\mu_{\nu },{\tau_{\nu }}^{-1}), & \log\;\sigma^{-2} & ∼\text{N}(\mu_{\sigma },{\tau_{\sigma }}^{-1})I_{\left[1,\infty \right]}. & \end{array}$$

Bayesian inference is carried out using relatively uninformative priors (see Table C.1 for prior hyper-parameter values). Lognormal prior distributions are chosen to ensure logistic growth and precision parameters are strictly positive. Ensuring that logistic growth parameters are positive disallows biologically unrealistic cultures, for example, a yeast population size cannot be negative so we do not expect the underlying process to have a negative carrying capacity *K*. In order to avoid unnecessary exploration of extremely low probability regions and to ensure that intrinsic noise does not dominate the process, we truncate our prior for log *σ*^−2^. We chose a lower limit of 1 after observing forward simulations from our processes, using logistic growth parameter values across a large parameter space (while covering representative parameter choices for microbial population growth curves, see Section 5.2) and increasing log *σ*^−2^ until intrinsic noise is so large that the deterministic part of the process is masked, rendering inference for the growth parameters impractical. An assumption of the LNA is that intrinsic noise is small and so it is natural to restrict large intrinsic noise.

The state space model for the LNAA is as follows:

(17) $$\begin{array}{cc} y_{t_{i}} & ∼\text{N}(X_{t_{i}},\nu^{2}),\\ \left(X_{t_{i}}|X_{t_{i-1}}=x_{t_{i-1}}\right) & ∼\text{N}(\mu_{t_{i}},\Xi_{t_{i}}),\;\mathrm{where}\;\;x_{t_{i}}=v_{t_{i}}+z_{t_{i}}, \end{array}$$

$\mu_{t_{i}}$ and $\Xi_{t_{i}}$ are given by (15). Priors are as in (16). Measurement error for the observed values is modelled with a normal distribution so that we have a linear Gaussian structure. The state space models in (16), (17) have different measurement error structures. To make a fair comparison between (16), (17), we chose our priors so that the marginal moments for the measurement error of our models are similar by visual inspection of simulated growth curves, paying particular attention to the exponential growth phase, where growth is fastest.

To see how the inference from our approximate models compares with slower “exact” models, we carry out inference using the approach of Golightly and Wilkinson (2005) with E-M simulations of (4) and of the log transformed process, using 15 evenly spaced intervals between simulated observations. We used a single site update algorithm to update model parameters and the E-M approximation of the latent process in turn. Given these simulations we can construct a state space model for an “exact” SLGM with lognormal measurement error (SLGM+L) and similarly for the SLGM with normal measurement error (SLGM+N), priors are as in (16).

We use the approach of Komorowski et al. (2009) to carry out inference for our approximate SDEs with the Kalman filter. Our inference makes use of a Kalman filter to integrate out the state process. The Kalman filer allows for fast inference compared to slow numerical simulation approaches that impute all states. The algorithm for our approximate models is the Metropolis-within-Gibbs sampler with a symmetric proposal (Gamerman and Lopes, 2006). Full-conditionals are sampled in turn to give samples from the joint posterior distribution. Each update in our algorithm is a Metropolis-Hastings step using a Kalman filter. Proposals are tuned for each update during a burn-in period. Faster inference could potentially be achieved by carrying out joint parameter updates. Posterior means are used as point estimates of parameter values and standard deviations are used to describe variation of inferred parameters. The Heidelberger and Welch convergence diagnostic (Heidelberger and Welch, 1981) is used to determine whether convergence has been achieved for all parameters.

**Fig. 2:**
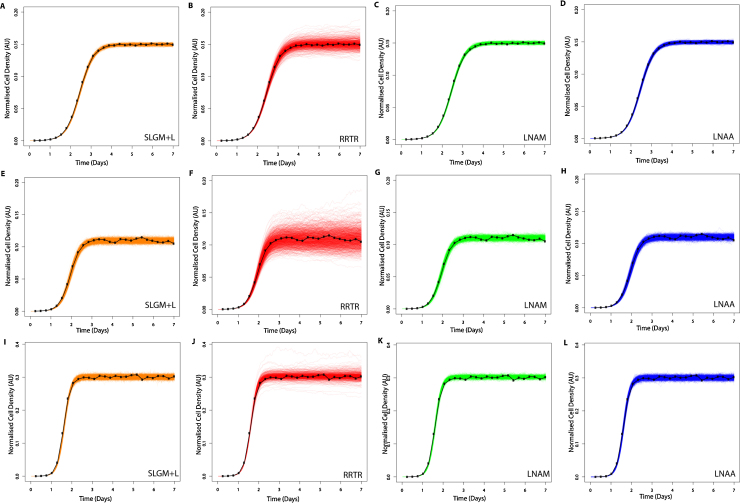
Forward trajectories with measurement error, simulated from parameter posterior samples (sample size = 1000). Model fitting is carried out on SLGM forward trajectories with lognormal measurement error (black), for three different sets of parameters (see Table 2). See (16) or (17) for model and Table C.1 for prior hyper-parameter values. Each row of figures corresponds to a different time course data set, simulated from a different set of parameter values, see Table 2. Each column of figures corresponds to a different model fit: (A), (E) and (I) SLGM+L (orange). (B), (F) and (J) RRTR model with lognormal error (red). (C), (G) and (K) LNAM model with lognormal error (green). (D), (H) and (L) LNAA model with normal error (blue). See Table 2 for parameter posterior means and true values. (For interpretation of the references to color in this figure legend, the reader is referred to the web version of the article.)

To compare our ability to recover SLGM parameters (with lognormal measurement error) using inference with LNA models, we simulate data and carry out Bayesian inference. Fig. 2 shows that all three approximate models can capture the synthetic time-course data well, but that the RRTR model is the least representative with the largest amount of drift occurring at the saturation stage, a property not found in the SLGM or the two new LNA models. Comparing forwards trajectories with measurement error (Fig. 2), the “exact” model is visually similar to our new approximate models. Further, Table 2 demonstrates that parameter posterior means are close to the true values and that standard deviations are small for all models and each parameter set. By comparing posterior means and standard deviations to the true values, Table 2 shows that all our models are able to recover the three different parameter sets considered.

**Fig. 3:**
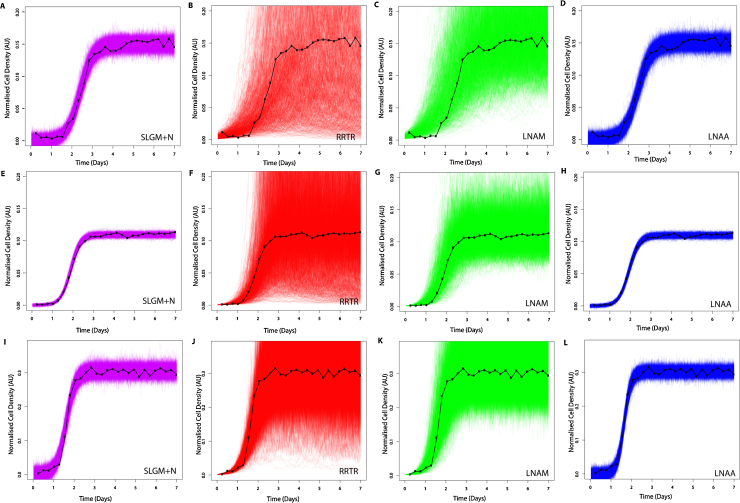
Forward trajectories with measurement error, simulated from inferred parameter posterior samples (sample size = 1000). Model fitting is carried out on SLGM forward trajectories with normal measurement error (black), for three different sets of parameters (see Table 2). See (16) or (17) for model and Table C.1 for prior hyper-parameter values. Each row of figures corresponds to a different time course data set, simulated from a different set of parameter values, see Table 2. Each column of figures corresponds to a different model fit: (A), (E) and (I) SLGM+N (pink). (B), (F) and (J) RRTR model with lognormal error (red). (C), (G) and (K) LNAM model with lognormal error (green). (D), (H) and (L) LNAA model with normal error (blue). See Table 2 for parameter posterior means and true values.(For interpretation of the references to color in this figure legend, the reader is referred to the web version of the article.)

**Fig. E.1:**
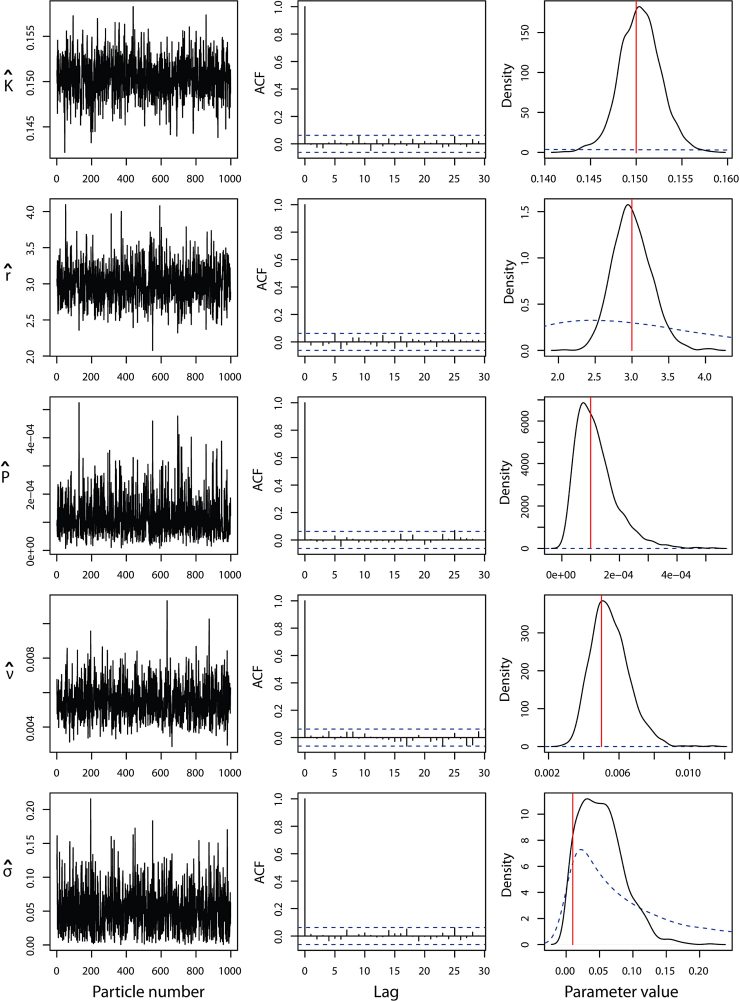
Trace, auto-correlation and density plots for the LNAA model parameter posteriors (sample size = 1000, thinning interval = 4000), see Fig. 3D. Posterior density (black), prior density (dashed blue) and true parameter values (red) are shown in the right hand column. (For interpretation of the references to color in this figure legend, the reader is referred to the web version of the article.)

**Fig. E.2:**
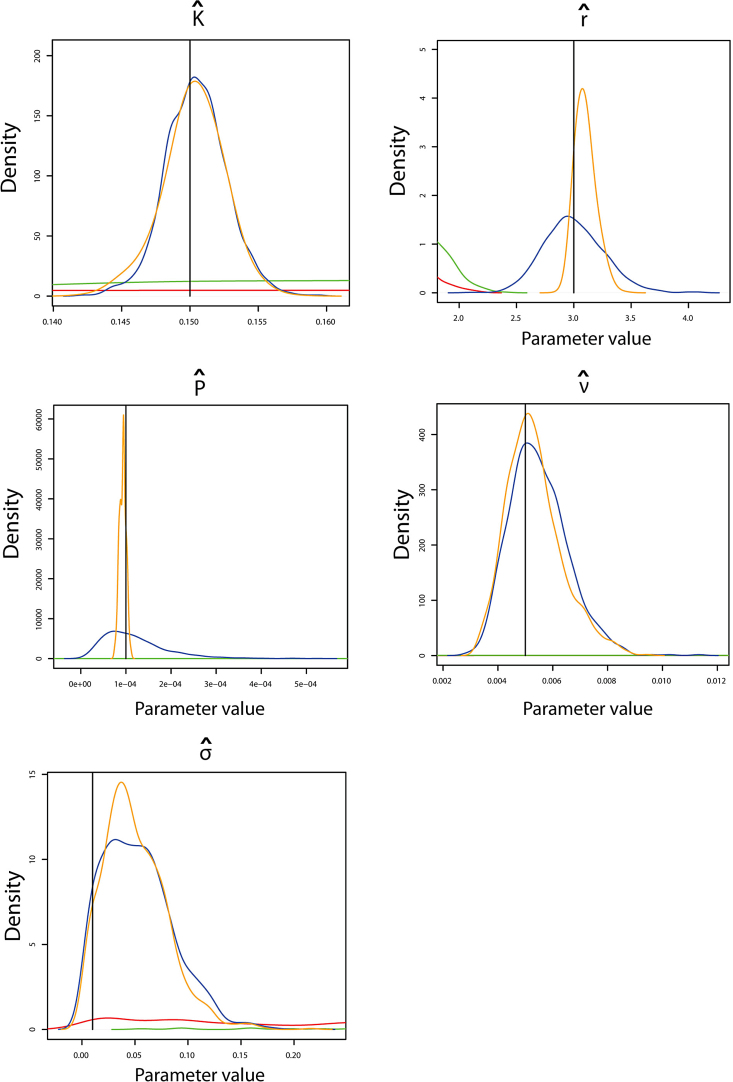
Density plots with overlaid curves for the SLGM (orange), RRTR (red), LNAM (green) and LNAA (blue) model parameter posteriors (sample size = 1000, thinning interval = 4000), see Fig. 3D. True parameter values are given in black. (For interpretation of the references to color in this figure legend, the reader is referred to the web version of the article.)

To compare our ability to recover SLGM parameters (with normal measurement error) using inference with LNA models, we simulate data and carry out Bayesian inference. Fig. 3 shows that, of our approximate models, only the LNAA model can appropriately represent the simulated time-courses as both models with lognormal measurement error: the RRTR and LNAM do not closely match the data. Comparing forward trajectories with measurement error (Fig. 3), the “exact” model is most visually similar to the LNAA, which shares the same measurement error structure. Further, Table 2 demonstrates that only our models with normal measurement error have posterior means close to the true values and that standard deviations are larger in the models with lognormal measurement error. Observing the posterior means for *K* and each parameter set (Table 2), we can see that the RRTR has the largest standard deviations and that, of the approximate models, its posterior means are furthest from both the true values and the “exact” model posterior means. Comparing LNA models to the “exact” models with matching measurement error, we can see in Table 2 that they share similar posterior means and only slightly larger standard deviations. Example posterior diagnostics given in Fig. E.1, demonstrate that posteriors are distributed tightly around true values for our LNAA and data from the SLGM with normal measurement error. Density plots with overlaid curves for the SLGM, RRTR, LNAM and LNAA model parameter posteriors used in Fig. 3D are given in Fig. E.2. Fig. E.2 also shows that inference using the LNAA gives parameter posteriors that are most similar to the SLGM and that posteriors from the LNAA have greater density over the true parameter values than other approximations.

### 5.2 Application to observed yeast data

**Fig. 4:**
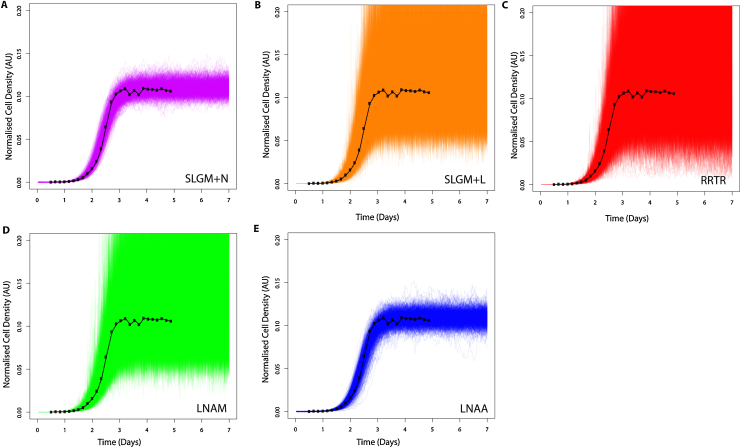
Forward trajectories with measurement error, simulated from inferred parameter posterior samples (sample size = 1000). Model fitting is carried out on observed yeast time-course data (black). See (16) or (17) and Table C.1 for prior hyper-parameter values. See Table 2 for parameter posterior means. (A) SLGM+N (pink). (B) SLGM+L (orange). (A) RRTR model with lognormal error (red). (B) LNAM model with lognormal error (green). (C) LNAA model with normal error (blue). (For interpretation of the references to color in this figure legend, the reader is referred to the web version of the article.)

We now consider which diffusion equation model can best represent observed microbial population growth curves taken from a quantitative fitness analysis (QFA) experiment (Addinall et al., 2011, Banks et al., 2012), see Fig. 4. The observed data consist of scaled cell density estimates over time for a population, or culture of budding yeast *Saccharomyces cerevisiae*. Independent replicate cultures are inoculated on plates and photographed over a period of 5 days. Captured images are then converted into estimates of integrated optical density (IOD, which we assume are proportional to cell population size), by the software package Colonyzer (Lawless et al., 2010). The dataset chosen for model fitting is a representative set of time-courses for 10 independent populations, each with 27 time points.

As in Fig. 3, we see that the LNAA model is the only approximation that can appropriately represent the time-course data. Both the RRTR and LNAM fail to bound the data as tightly as the LNAA (Fig. 4). Our two “exact” models are visually similar to our approximate models with the same measurement error. The SLGM+N is most similar to the LNAA and the SLGM+L is most similar to the RRTR and LNAM. This is as expected due to matching measurement error structures. Table 2 summarises parameter estimates for the observed yeast data using each model. The variation in the LNAA model parameter posteriors is much smaller than the RRTR and LNAM, indicating a more appropriate model fit. Comparing the LNA models and “exact” models with matching measurement error, we can see in Table 2 that they share similar posterior means and standard deviations for all parameters and in particular, they are very similar for both *K* and *r*, which are important phenotypes for calculating fitness (Addinall et al., 2011).

In Table 3, we compare the quality of parameter inference for 10 observed yeast time-courses with each approximate model. MSEs for 1000 posterior sample forward simulations are calculated for each yeast time course and summed to give a Total MSE for each model. The RRTR has the highest total MSE and a much larger total MSE than the “exact” SLGM+L. It is interesting to see there is a very similar total MSE for the SLGM+L and LNAM, and similarly for the SLGM+N and LNAA, demonstrating that our approximations perform well.

**Table 3:** Total mean squared error (MSE) for 10 observed yeast growth time courses, each with 1000 forward simulated time-courses with measurement error. Parameter values are taken from posterior samples. Standard Deviations give the variation between the sub-total MSEs for each yeast time course fit.

Model	SLGM+N	SLGM+L	RRTR	LNAM	LNAA
Total MSE	29.847	100.165	600.601	99.397	30.959
Standard deviation	1.689	8.391	55.720	9.263	2.030

Computational times for convergence of our MCMC schemes (code is available at http://github.com/jhncl/LNA.git) can be compared using estimates for the minimum effective sample size per second (ESS_min_/sec) (Plummer et al., 2006). Table F.1 shows the ESS_min_/sec of the approximate models compared to the exact approaches. The average ESS_min_/sec of our approximate model (coded in C) is ∼100 and “exact” model ∼1 (coded in JAGS (Plummer, 2010) with 15 imputed states between time points, chosen to maximise ESS_min_/s). Our software for inference (coded in C) is about twice as fast as the simple MCMC scheme used by JAGS, indicating that our inference is ∼50× faster than an “exact” approach. A more efficient “exact” approach could speed up further, say by another factor of 5, but our approximate approach will remain at least an order of magnitude faster (and the approximate approach could also be speeded up with a little work). To ensure convergence of our Gibbs sampler we use a burn-in of 600,000 and a thinning of 4000 to obtain a final posterior sample size of 1000. Our approximate models describe the mean curve of the SLGM well, when carrying out inference for models of systems with more complicated underlying dynamics, the restarting method of Fearnhead et al. (2014) could be used to give improved approximation and increased numerical stability.

## 6 Conclusion

We have presented two new diffusion processes for modelling logistic growth data where fast inference is required: the linear noise approximation (LNA) of the stochastic logistic growth model (SLGM) with multiplicative noise and the LNA of the SLGM with additive intrinsic noise. Both the LNAM and LNAA are derived from the linear noise approximation of the stochastic logistic growth model (SLGM). The new diffusion processes approximate the SLGM more closely than an alternative approximation (RRTR) proposed by Román-Román and Torres-Ruiz (2012). The RRTR lacks a mean reverting property that is found in the SLGM, LNAM and LNAA, resulting in increasing variance during the stationary phase of population growth (see Fig. 1). The likelihood for a state space model with either the RRTR and LNAM is only tractable with lognormal measurement error. The LNAA differs as the likelihood is only tractable with normal measurement error. We are therefore able to cover two types of measurement error, with analytical solutions to the LNA and a tractable likelihood.

We compared the ability of the LNAM, LNAA and RRTR to approximate the SLGM by recovering parameter values from simulated datasets using standard MCMC techniques. When modelling stochastic logistic growth with lognormal measurement error we find that our approximate models are able to represent data simulated from the original process.When modelling stochastic logistic growth with normal measurement error we find that only our models with normal measurement error can appropriately track data simulated from the original process (see Fig. 3). We also compared parameter posterior distribution summaries with parameter values used to generate simulated data after inference using both approximate and “exact” models (see Table 2). We find that, when using the RRTR model, posterior distributions for the carrying capacity parameter *K* are less precise than for the LNAM and LNAA approximations. We also note that it is not possible to model normal measurement error while maintaining a linear Gaussian structure (which allows fast inference with the Kalman filter) when carrying out inference with the RRTR. We conclude that for additive measurement error, the LNAA model is the most appropriate approximate model.

To test model performance during inference with real population data, we fitted our approximate models and the “exact” SLGM to microbial population growth curves generated by quantitative fitness analysis (QFA) (see Fig. 4). We found that the LNAA model was the most appropriate for modelling experimental data. It seems likely that this is because a normal error structure best describes this particular dataset, placing the LNAM and RRTR models at a disadvantage. We demonstrate that arbitrarily exact methods and our fast approximations perform similarly during inference for 10 diverse, experimentally observed, microbial population growth curves (see Table 3) which shows that, in practise, our fast approximations are as good as “exact” methods.

It is interesting to note that, although the LNAA is not a better approximation of the original SGLM process than the LNAM, it is still quite reasonable. Figs. 1A and D shows that the SLGM and LNAA processes are visually similar. Fig. 1E demonstrates that forward trajectories of the LNAA also share similar levels of variation over time with the SLGM and LNAM.

Fast inference with the LNAA gives us the potential to develop large hierarchical Bayesian models which simultaneously describe thousands of independent time-courses from QFA with a diffusion equation, allowing us to infer the existence of genetic interactions on a genome-wide scale using realistic computational resources.

Here, we have concentrated on a biological model of population growth. However, we expect that the approach we have demonstrated: generating linear noise approximations of stochastic processes to allow fast Bayesian inference with Kalman filtering for marginal likelihood computation, will be useful in a wide range of other applications where simulation is prohibitively slow.

## Figures and Tables

**Table C.1:** Prior hyper-parameters for Bayesian sate space models, Lognormal with mean (*μ*) and precision (*τ*)

Parameter name	Value
*μ*_*K*_	log(0.1)
*τ*_*K*_	2
*μ*_*r*_	log(3)
*τ*_*r*_	5
*μ*_*P*_	log(0.0001)
*τ*_*P*_	0.1
*μ*_*σ*_	log(100)
*τ*_*σ*_	0.1
*μ*_*ν*_	log(10000)
*τ*_*ν*_	0.1

**Table F.1:** Average minimum effective sample size per second (*ESS*_*min*_/*sec*) and average CPU time in seconds for the SLGM+N, SLGM+L, RRTR, LNAM and LNAA for 10 model applications to observed yeast data in Fig. 4 (excluding burn-in time). A sample size of 600,000 without thinning is obtained after a burn-in of 600,000.

Model	SLGM+N	SLGM+L	RRTR	LNAM	LNAA
Average *ESS*_*min*_/*s*	1.11	0.87	74.90	78.66	121.01
Average CPU time (s)	2397	2463	187	188	195

## Appendix A LNAM solution

First we look to solve *dZ*_*t*_, given in Eq. (11). We define $f(t)=-{\mathrm{be}}^{v_{t}}=-\frac{{\mathrm{baPe}}^{{\mathrm{at}}_{i}}}{\mathrm{bP}(e^{{\mathrm{at}}_{i}}-1)+a}$ to obtain the following,

$${\mathrm{dZ}}_{t}=f(t)Z_{t}\mathrm{dt}+\sigma {\mathrm{dW}}_{t}.$$

Conditioning on *Z*_0_, the solution of *Z*_*t*_ has the following form (Arnold, 2013):

$$Z_{t}=\phi (t)\left[Z_{0}+\int_{0}^{t}\phi {\left(s\right)}^{-1}\sigma {\mathrm{dW}}_{s}\right],$$

where $\phi (t)=e^{\int_{0}^{t}f(s)\mathrm{ds}}=\frac{a}{\mathrm{bP}(e^{{\mathrm{at}}_{i}}-1)+a}$.

Note that the solution to the above integral is given by

$$\int_{0}^{t}f(s)\mathrm{ds}=\int_{0}^{t}-\frac{{\mathrm{baPe}}^{\mathrm{as}}}{\mathrm{bP}(e^{\mathrm{as}}-1)+a}\mathrm{ds}=\log\left(\frac{a}{\mathrm{bP}(e^{{\mathrm{at}}_{i}}-1)+a}\right).$$

Finally, the distribution at time *t* is *Z*_*t*_|*Z*_0_ ∼ *N*(*M*_*t*_, *E*_*t*_) (Arnold, 2013), where

*M*_*t*_ = *ϕ*(*t*)*Z*_0_ and $E_{t}=\phi {\left(t\right)}^{2}\int_{0}^{t}{\left[{\phi (s)}^{-1}\sigma \right]}^{2}\mathrm{ds}$.

Further, $M_{t}=\frac{a}{\mathrm{bP}(e^{{\mathrm{at}}_{i}}-1)+a}Z_{0}$ and $E_{t}=\sigma^{2}{\left[(a/\mathrm{bP}(e^{{\mathrm{at}}_{i}}-1)+a)\right]}^{2}\int_{0}^{t}{\left[(a/\mathrm{bP}(e^{\mathrm{as}}-1)+a)\right]}^{-2}$ds.

As $\int_{0}^{t}{\left[\frac{a}{\mathrm{bP}(e^{\mathrm{as}}-1)+a}\right]}^{-2}\mathrm{ds}=\frac{b^{2}P^{2}e^{2{\mathrm{at}}_{i}}+4\mathrm{bP}(a-\mathrm{bP})e^{{\mathrm{at}}_{i}}+2\mathrm{at}{\left(a-\mathrm{bP}\right)}^{2}}{2a^{3}}-\frac{b^{2}P^{2}+4\mathrm{bP}(a-\mathrm{bP})}{2a^{3}}$,

$$\begin{array}{cc} E_{t}= & \sigma^{2}{\left[\frac{a}{\mathrm{bP}(e^{{\mathrm{at}}_{i}}-1)+a}\right]}^{2}\left[\frac{b^{2}P^{2}(e^{2{\mathrm{at}}_{i}}-1)+4\mathrm{bP}(a-\mathrm{bP})(e^{{\mathrm{at}}_{i}}-1)+2{\mathrm{at}}_{i}{\left(a-\mathrm{bP}\right)}^{2}}{2a^{3}}\right]\\ = & \sigma^{2}\left[\frac{b^{2}P^{2}(e^{2{\mathrm{at}}_{i}}-1)+4\mathrm{bP}(a-\mathrm{bP})(e^{{\mathrm{at}}_{i}}-1)+2{\mathrm{at}}_{i}{\left(a-\mathrm{bP}\right)}^{2}}{2a{\left(\mathrm{bP}(e^{{\mathrm{at}}_{i}}-1)+a\right)}^{2}}\right]. \end{array}$$

Taking our solutions for $v_{t}$ (10) and *Z*_*t*_, we can now write our solution for the LNA to the log of the logistic growth process (8).

As $Y_{t}=v_{t}+Z_{t}$,

$$Y_{t}|Y_{0}∼N\left(\log\left[\frac{{\mathrm{aPe}}^{{\mathrm{at}}_{i}}}{\mathrm{bP}(e^{{\mathrm{at}}_{i}}-1)+a}\right]+M_{t},E_{t}\right).$$

Note: $\frac{{\mathrm{aPe}}^{{\mathrm{at}}_{i}}}{\mathrm{bP}(e^{{\mathrm{at}}_{i}}-1)+a}$ has the same functional form as the solution to the deterministic part of the logistic growth process (4) and is equivalent when *σ* = 0 (such that $a=r-\frac{\sigma^{2}}{2}=r$).

Further, as *Y*_*t*_ is normally distributed, we know *X*_*t*_ = *e*^*Y*_*t*_^ will be log normally distributed and

$$X_{t}|X_{0}∼\log\;N(\log\left(\frac{{\mathrm{aPe}}^{{\mathrm{at}}_{i}}}{\mathrm{bP}(e^{{\mathrm{at}}_{i}}-1)+a}\right)+M_{t},E_{t}).$$

Alternatively set *Q* = (*a*/*bP* − 1),

$$X_{t}|X_{0}∼\log\;N(\log\left(\frac{\frac{a}{b}}{1+{\mathrm{Qe}}^{-\mathrm{at}}}\right)+M_{t},E_{t}).$$

From our solution to the log process we can obtain the following transition density

$$\begin{array}{cc} \begin{array}{cc} (Y_{t_{i}}|Y_{t_{i-1}} & =y_{t_{i-1}})∼\text{N}\left(\mu_{t_{i}},\Xi_{t_{i}}\right),\\ \mathrm{where}\;y_{t_{i-1}} & =v_{t_{i-1}}+z_{t_{i-1}},Q=\left(\frac{\frac{a}{b}}{P}-1\right)e^{{\mathrm{at}}_{0}},\\ \mu_{t_{i}} & =y_{t_{i-1}}+\log\left(\frac{1+{\mathrm{Qe}}^{-{\mathrm{at}}_{i-1}}}{1+{\mathrm{Qe}}^{-{\mathrm{at}}_{i}}}\right)+e^{-a(t_{i}-t_{i-1})}\frac{1+{\mathrm{Qe}}^{-{\mathrm{at}}_{i-1}}}{1+{\mathrm{Qe}}^{-{\mathrm{at}}_{i}}}z_{t_{i-1}}\;\mathrm{and}\\ \Xi_{t_{i}} & =\sigma^{2}\left[\frac{4Q(e^{{\mathrm{at}}_{i}}-e^{{\mathrm{at}}_{i-1}})+e^{2{\mathrm{at}}_{i}}-e^{2{\mathrm{at}}_{i-1}}+2{\mathrm{aQ}}^{2}(t_{i}-t_{i-1})}{2a{\left(Q+e^{{\mathrm{at}}_{i}}\right)}^{2}}\right]. \end{array} & \end{array}$$

In order to match our initial conditions correctly, we set *Z*_0_ = 0.

## Appendix B LNAA solution

First we look to solve *dZ*_*t*_, given in (14). We define *f*(*t*) = *a* − 2*bv*_*t*_ to obtain the following,

$${\mathrm{dZ}}_{t}=f(t)Z_{t}\mathrm{dt}+\sigma v_{t}{\mathrm{dW}}_{t}.$$

Conditioning on *Z*_0_, the solution of *Z*_*t*_ has the following form (Arnold, 2013):

$$Z_{t}=\phi (t)\left[Z_{0}+\int_{0}^{t}\phi {\left(s\right)}^{-1}\sigma {\mathrm{dW}}_{s}\right],$$

where $\phi (t)=e^{\int_{0}^{t}f(s)\mathrm{ds}}=e^{{\mathrm{at}}_{i}}{\left(\frac{a}{\mathrm{bP}(e^{{\mathrm{at}}_{i}}-1)+a}\right)}^{2}$.

Note that the solution to the above integral is given by

$$\int_{0}^{t}f(s)\mathrm{ds}=\int_{0}^{t}(a-2{\mathrm{bV}}_{s})\mathrm{ds}={\mathrm{at}}_{i}-2\log\left(\frac{\mathrm{bP}(e^{{\mathrm{at}}_{i}}-1)+a}{a}\right),$$

where $\int_{0}^{t}V_{s}\mathrm{ds}=\frac{1}{b}\log\left(\frac{\mathrm{bP}(e^{{\mathrm{at}}_{i}}-1)+a}{a}\right)$.

Finally the distribution at time *t* is *Z*_*t*_|*Z*_0_ ∼ *N*(*M*_*t*_, *E*_*t*_) (Arnold, 2013), where

*M*_*t*_ = *ϕ*(*t*)*Z*_0_ and $E_{t}=\phi {\left(t\right)}^{2}\int_{0}^{t}{\left[{\phi (s)}^{-1}\sigma \right]}^{2}\mathrm{ds}$.

$$M_{t}=e^{{\mathrm{at}}_{i}}{\left(\frac{a}{\mathrm{bP}(e^{{\mathrm{at}}_{i}}-1)+a}\right)}^{2}Z_{0}$$

and

$$E_{t}={\left(e^{{\mathrm{at}}_{i}}{\left(\frac{a}{\mathrm{bP}(e^{{\mathrm{at}}_{i}}-1)+a}\right)}^{2}\right)}^{2}\int_{0}^{t}{\left[e^{\mathrm{as}}{\left(\frac{a}{\mathrm{bP}(e^{\mathrm{as}}-1)+a}\right)}^{2}\right]}^{-2}\sigma^{2}V_{s}^{2}\mathrm{ds}$$

$$=\sigma^{2}{\left(e^{{\mathrm{at}}_{i}}{\left(\frac{a}{\mathrm{bP}(e^{{\mathrm{at}}_{i}}-1)+a}\right)}^{2}\right)}^{2}\int_{0}^{t}{\left[e^{\mathrm{as}}{\left(\frac{a}{\mathrm{bP}(e^{\mathrm{as}}-1)+a}\right)}^{2}\right]}^{-2}{\left[\frac{{\mathrm{aPe}}^{\mathrm{as}}}{\mathrm{bP}(e^{\mathrm{as}}-1)+a}\right]}^{2}\mathrm{ds}.$$

$$=\sigma^{2}{\left(e^{{\mathrm{at}}_{i}}{\left(\frac{a}{\mathrm{bP}(e^{{\mathrm{at}}_{i}}-1)+a}\right)}^{2}\right)}^{2}\int_{0}^{t}\left[e^{-2\mathrm{as}}{\left(\frac{a}{\mathrm{bP}(e^{\mathrm{as}}-1)+a}\right)}^{-4}\right]{\left[\frac{{\mathrm{aPe}}^{\mathrm{as}}}{\mathrm{bP}(e^{\mathrm{as}}-1)+a}\right]}^{2}\mathrm{ds}$$

$$=\sigma^{2}{\left(e^{{\mathrm{at}}_{i}}{\left(\frac{1}{\mathrm{bP}(e^{{\mathrm{at}}_{i}}-1)+a}\right)}^{2}\right)}^{2}\int_{0}^{t}\left[a^{2}P^{2}{\left(\frac{1}{\mathrm{bP}(e^{\mathrm{as}}-1)+a}\right)}^{-2}\right]\mathrm{ds},$$

as $\int_{0}^{t}{\left(\frac{1}{\mathrm{bP}(e^{\mathrm{as}}-1)+a}\right)}^{-2}\mathrm{ds}=\frac{b^{2}P^{2}e^{2{\mathrm{at}}_{i}}+4\mathrm{bP}(a-\mathrm{bP})e^{{\mathrm{at}}_{i}}+2\mathrm{at}{\left(a-\mathrm{bP}\right)}^{2}}{2a}-\frac{b^{2}P^{2}+4\mathrm{bP}(a-\mathrm{bP})}{2a}$,

$$\begin{array}{cc} E_{t}= & \frac{1}{2}\sigma^{2}{\mathrm{aP}}^{2}e^{2{\mathrm{at}}_{i}}{\left(\frac{1}{\mathrm{bP}(e^{{\mathrm{at}}_{i}}-1)+a}\right)}^{4}\\ & \times \left[b^{2}P^{2}(e^{2{\mathrm{at}}_{i}}-1)+4\mathrm{bP}(a-\mathrm{bP})(e^{{\mathrm{at}}_{i}}-1)+2{\mathrm{at}}_{i}{\left(a-\mathrm{bP}\right)}^{2}\right]. \end{array}$$

Taking our solutions for $v_{t}$ (13) and *Z*_*t*_, we can obtain the following transition density

$$\begin{array}{cc} \begin{array}{cc} (X_{t_{i}}|X_{t_{i-1}}= & x_{t_{i-1}})∼N(\mu_{t_{i}},\Xi_{t_{i}}),\\ \mathrm{where}\;x_{t_{i-1}}= & v_{t_{i-1}}+z_{t_{i-1}},\\ \mu_{t_{i}}= & x_{t_{i-1}}+\left(\frac{{\mathrm{aPe}}^{{\mathrm{at}}_{i}}}{\mathrm{bP}(e^{{\mathrm{at}}_{i}}-1)+a}\right)-\left(\frac{{\mathrm{aPe}}^{{\mathrm{at}}_{i-1}}}{\mathrm{bP}(e^{{\mathrm{at}}_{i-1}}-1)+a}\right)\\ & +e^{a(t_{i}-t_{i-1})}{\left(\frac{\mathrm{bP}(e^{{\mathrm{at}}_{i-1}}-1)+a}{\mathrm{bP}(e^{{\mathrm{at}}_{i}}-1)+a}\right)}^{2}Z_{t_{i-1}}\;\mathrm{and}\\ \Xi_{t_{i}}= & \frac{1}{2}\sigma^{2}{\mathrm{aP}}^{2}e^{2{\mathrm{at}}_{i}}{\left(\frac{1}{\mathrm{bP}(e^{{\mathrm{at}}_{i}}-1)+a}\right)}^{4}\\ & \times [b^{2}P^{2}(e^{2{\mathrm{at}}_{i}}-e^{2{\mathrm{at}}_{i-1}})+4\mathrm{bP}(a-\mathrm{bP})(e^{{\mathrm{at}}_{i}}-e^{{\mathrm{at}}_{i-1}})\\ & \;+2a(t_{i}-t_{i-1}){\left(a-\mathrm{bP}\right)}^{2}]. \end{array} & \end{array}$$

In order to match our initial conditions correctly, we set *Z*_0_ = 0.

## Appendix C Prior hyper-parameters for Bayesian state space models

See Table C.1

## Appendix D Kalman filter

To obtain $\pi (y_{t_{1:N}})$ for the LNAA with normal measurement error we can use the following Kalman Filter algorithm. $\theta_{t_{i}}$ and $Y_{t_{i}}$ are the state and measurement processes respectively. $w_{t}$ and *u*_*t*_ is the state and measurement error respectively, where $w_{t}$ and *u*_*t*_ are IID, $E[w_{t}]=0$, *E*[*u*_*t*_] = 0, $E[w_{t}{w_{t}}^{T}]=W_{t}$ and *E*[*u*_*t*_*u*_*t*_^*T*^] = *U* (measurement error is not time dependent). The unobserved latent process is driven by

$$\theta_{t_{i}}|\theta_{t_{i-1}}∼\text{N}(G_{t_{i}}\theta_{t_{i-1}},W_{t_{i}})$$

and the measurement error distribution, relating the latent variable to the observed is given by,

$$Y_{t_{i}}|\theta_{t_{i}}∼\text{N}(F_{t_{i}}^{T}\theta_{t_{i}},U),$$

where *U* and matrices $F_{t_{i}}$, $G_{t_{i}}$ and $W_{t_{i}}$ are all given. The Kalman filter consists of the recursion,

$$\begin{array}{cc} \theta_{t_{i}}|y_{1:t_{i}} & ∼\text{N}(m_{t_{i}},C_{t_{i}}),\\ m_{t_{i}} & =a_{t_{i}}+R_{t_{i}}F{\left(F^{T}R_{t_{i}}F+U\right)}^{-1}[y_{t_{i}}-F^{T}a_{t_{i}}],\\ C_{t_{i}} & =R_{t_{i}}-R_{t_{i}}F{\left(F^{T}R_{t_{i}}F+U\right)}^{-1}F^{T}R_{t_{i}} \end{array}$$

initialized with *m*_0_ = *P* and *C*_0_ = 0, where

$$\begin{array}{cc} \theta_{t_{i}}|y_{1:t_{i-1}} & ∼\text{N}(a_{t_{i}},R_{t_{i}}),\\ a_{t_{i}} & =G_{t_{i}}m_{t_{i-1}}\\ \text{and}\;R_{t_{i}} & =G_{t_{i}}C_{t_{i-1}}G_{t_{i}}^{T}+W_{t_{i}}. \end{array}$$

See (West and Harrison, 1997) for further details.

The transition density distribution, see (15) is as follows:

$$\begin{array}{cc} \theta_{t_{i}}|\theta_{t_{i-1}} & ∼\text{N}(G_{t_{i}}\theta_{t_{i-1}},W_{t_{i}})\\ \mathrm{or}\mathrm{equivalently}\;(X_{t_{i}}|X_{t_{i-1}}=x_{t_{i-1}}) & ∼\text{N}\left(\mu_{t_{i}},\Xi_{t_{i}}\right),\;\mathrm{where}\;x_{t_{i-1}}=v_{t_{i-1}}+z_{t_{i-1}},\\ \theta_{t} & =\left(\begin{array}{c} 1\\ X_{t_{i}} \end{array}\right)=\left(\begin{array}{cc} 1 & 0\\ H_{\alpha ,t_{i}} & H_{\beta ,t_{i}} \end{array}\right)\left(\begin{array}{c} 1\\ X_{t_{i-1}} \end{array}\right)\\ & =G_{t_{i}}\theta_{t_{i-1}},\\ G_{t_{i}} & =\left(\begin{array}{cc} 1 & 0\\ H_{\alpha ,t_{i}} & H_{\beta ,t_{i}} \end{array}\right),\;W_{t_{i}}=\left(\begin{array}{cc} 0 & 0\\ 0 & \Xi_{t_{i}} \end{array}\right)\\ \mathrm{where}\;H_{\alpha ,t_{i}}=H_{\alpha }(t_{i},t_{i-1})= & v_{t}-V_{t-1}e^{a(t_{i}-t_{i-1})}{\left(\frac{\mathrm{bP}(e^{{\mathrm{aT}}_{i-1}}-1)+a}{\mathrm{bP}(e^{{\mathrm{aT}}_{i}}-1)+a}\right)}^{2}\\ \mathrm{and}\;H_{\beta ,t_{i}}= & H_{\beta }(t_{i},t_{i-1})=e^{a(t_{i}-t_{i-1})}{\left(\frac{\mathrm{bP}(e^{{\mathrm{aT}}_{i-1}}-1)+a}{\mathrm{bP}(e^{{\mathrm{aT}}_{i}}-1)+a}\right)}^{2}. \end{array}$$

The measurement error distribution is as follows:

$$\begin{array}{cc} y_{t_{i}}|\theta_{t_{i}}∼ & \text{N}(F^{T}\theta_{t_{i}},U)\\ \text{or equivalently}\;y_{t_{i}}|\theta_{t_{i}}∼ & \text{N}(X_{t_{i}},\sigma_{\nu }^{2}),\\ \mathrm{where}\;F= & \left(\begin{array}{c} 0\\ 1 \end{array}\right)\;\mathrm{and}\;U=\sigma_{\nu }^{2}. \end{array}$$

Matrix Algebra:

$$\begin{array}{cc} a_{t_{i}}= & G_{t_{i}}m_{t_{i-1}}\\ = & \left(\begin{array}{cc} 1 & 0\\ H_{\alpha ,t_{i}} & H_{\beta ,t_{i}} \end{array}\right)\left(\begin{array}{c} 1\\ m_{t_{i-1}} \end{array}\right)=\left(\begin{array}{c} 1\\ H_{\alpha ,t_{i}}+H_{\beta ,t_{i}}m_{t_{i-1}} \end{array}\right) \end{array}$$

$$\begin{array}{cc} R_{t_{i}} & =G_{t_{i}}C_{t_{i-1}}G_{t_{i}}^{T}+W_{t_{i}}\\ & =\left(\begin{array}{cc} 0 & 0\\ 0 & {H_{\beta ,t_{i}}}^{2}c_{t_{i-1}}^{2} \end{array}\right)+\left(\begin{array}{cc} 0 & 0\\ 0 & \Xi_{t_{i}} \end{array}\right)=\left(\begin{array}{cc} 0 & 0\\ 0 & {H_{\beta ,t_{i}}}^{2}c_{t_{i-1}}^{2}+\Xi_{t_{i}} \end{array}\right) \end{array}$$

$$\begin{array}{cc} C_{t_{i-1}} & =\left(\begin{array}{cc} 0 & 0\\ 0 & c_{t_{i-1}}^{2} \end{array}\right) \end{array}$$

$$\begin{array}{cc} R_{t_{i}}F{\left(F^{T}R_{t_{i}}F+U\right)}^{-1}= & \left(\begin{array}{cc} 0 & 0\\ 0 & {H_{\beta ,t_{i}}}^{2}c_{t_{i-1}}^{2}+\Xi_{t_{i}} \end{array}\right)\left(\begin{array}{c} 0\\ 1 \end{array}\right)\\ & \times {\left[\left(\begin{array}{cc} 0 & 1 \end{array}\right)\left(\begin{array}{cc} 0 & 0\\ 0 & {H_{\beta ,t_{i}}}^{2}c_{t_{i-1}}^{2}+\Xi_{t_{i}} \end{array}\right)\left(\begin{array}{c} 0\\ 1 \end{array}\right)+\sigma_{\nu }^{2}\right]}^{-1}\\ = & {\left[\left(\begin{array}{c} {H_{\beta ,t_{i}}}^{2}c_{t_{i-1}}^{2}+\Xi_{t_{i}}+\sigma_{\nu }^{2} \end{array}\right)\right]}^{-1}\left(\begin{array}{c} 0\\ {H_{\beta ,t_{i}}}^{2}c_{t_{i-1}}^{2}+\Xi_{t_{i}} \end{array}\right) \end{array}$$

$$\begin{array}{cc} m_{t_{i}}= & a_{t_{i}}+R_{t_{i}}F{\left(F^{T}R_{t_{i}}F+U\right)}^{-1}[y_{t_{i}}-F^{T}a_{t_{i}}]\\ = & \left(\begin{array}{c} 1\\ H_{\alpha ,t_{i}}+H_{\beta ,t_{i}}m_{t_{i-1}} \end{array}\right)\\ & +{\left[\left(\begin{array}{c} {H_{\beta ,t_{i}}}^{2}c_{t_{i-1}}^{2}+\Xi_{t_{i}}+\sigma_{\nu }^{2} \end{array}\right)\right]}^{-1}\left(\begin{array}{c} 0\\ {H_{\beta ,t_{i}}}^{2}c_{t_{i-1}}^{2}+\Xi_{t_{i}} \end{array}\right)\left[y_{t_{i}}-\left(\begin{array}{cc} 0 & 1 \end{array}\right)\left(\begin{array}{c} 1\\ H_{\alpha ,t_{i}}+H_{\beta ,t_{i}}m_{t_{i-1}} \end{array}\right)\right]\\ = & \left(\begin{array}{c} 0\\ H_{\alpha ,t_{i}}+H_{\beta ,t_{i}}m_{t_{i-1}}+\frac{{H_{\beta ,t_{i}}}^{2}c_{t_{i-1}}^{2}+\Xi_{t_{i}}}{{H_{\beta ,t_{i}}}^{2}c_{t_{i-1}}^{2}+\Xi_{t_{i}}+\sigma_{\nu }^{2}}\left[y_{t_{i}}-H_{\alpha ,t_{i}}-H_{\beta ,t_{i}}m_{t_{i-1}}\right] \end{array}\right) \end{array}$$

$$\begin{array}{cc} C_{t_{i}}= & R_{t_{i}}-R_{t_{i}}F{\left(F^{T}R_{t_{i}}F+U\right)}^{-1}F^{T}R_{t_{i}}\\ = & \left(\begin{array}{cc} 0 & 0\\ 0 & {H_{\beta ,t_{i}}}^{2}c_{t_{i-1}}^{2}+\Xi_{t_{i}} \end{array}\right)\\ & -{\left[\left(\begin{array}{c} {H_{\beta ,t_{i}}}^{2}c_{t_{i-1}}^{2}+\Xi_{t_{i}}+\sigma_{\nu }^{2} \end{array}\right)\right]}^{-1}\left(\begin{array}{c} 0\\ {H_{\beta ,t_{i}}}^{2}c_{t_{i-1}}^{2}+\Xi_{t_{i}} \end{array}\right)\left[\left(\begin{array}{cc} 0 & 1 \end{array}\right)\left(\begin{array}{cc} 0 & 0\\ 0 & {H\beta ,t_{i}}^{2}c_{t_{i-1}}^{2}+\Xi_{t_{i}} \end{array}\right)\right]\\ = & \left(\begin{array}{cc} 0 & 0\\ 0 & {H_{\beta ,t_{i}}}^{2}c_{t_{i-1}}^{2}+\Xi_{t_{i}}-\frac{{\left({H_{\beta ,t_{i}}}^{2}c_{t_{i-1}}^{2}+\Xi_{t_{i}}\right)}^{2}}{{H_{\beta ,t_{i}}}^{2}c_{t_{i-1}}^{2}+\Xi_{t_{i}}+\sigma_{\nu }^{2}} \end{array}\right) \end{array}$$

With $m_{t_{i}}$ and $C_{t_{i}}$ for *i* = 1 : *N*, we can evaluate $a_{t_{i}}$, $R_{t_{i}}$ and $\pi (x_{t_{i}}|y_{t_{1:(i-1)}})$ for *i* = 1 : *N*. We are interested in $\pi (y_{t_{1:i}})=\prod_{i=1}^{N}\pi (y_{t_{i}}|y_{t_{1:(i-1)}})$, where $\pi (y_{t_{i}}|y_{t_{1:(i-1)}})=\int_{x}\pi (y_{t_{i}}|x_{t_{i}})\pi (x_{t_{i}}|y_{t_{1:(i-1)}}){\mathrm{dx}}_{t_{i}}$ gives a tractable Gaussian integral. Finally,

$$\begin{array}{cc} \log\pi (y_{t_{1:N}}) & =\sum_{i=1}^{N}\log\pi (y_{t_{i}}|y_{t_{1:(i-1)}})\\ & =\sum_{i=1}^{N}\left[-\log\left(\sqrt{2\pi (\sigma_{f}^{2}+\sigma_{g}^{2})}\right)-\frac{{\left(\mu_{f}-\mu_{g}\right)}^{2}}{2(\sigma_{f}^{2}+\sigma_{g}^{2})}\right], \end{array}$$

$$\begin{array}{cc} \mathrm{where}\;\mu_{f}-\mu_{g}= & y_{t_{i}}-a_{t_{i}}=y_{t_{i}}-H_{\alpha ,t_{i}}-H_{\beta ,t_{i}}m_{t_{i-1}}\\ \mathrm{and}\;\sigma_{f}^{2}+\sigma_{g}^{2}= & \sigma_{\nu }^{2}+R_{t_{i}}=\sigma_{\nu }^{2}+{H_{\beta ,t_{i}}}^{2}c_{t_{i-1}}^{2}+\Xi_{t_{i}}. \end{array}$$

Procedure

1 Set *i* = 1. Initialize *m*_0_ = *P* and *C*_0_ = 0.
2 Evaluate and store the following log likelihood term:
  $$\begin{array}{cc} \log\pi (y_{t_{i}}|y_{t_{1:(i-1)}})= & \left[-\log\left(\sqrt{2\pi (\sigma_{f}^{2}+\sigma_{g}^{2})}\right)-\frac{{\left(\mu_{f}-\mu_{g}\right)}^{2}}{2(\sigma_{f}^{2}+\sigma_{g}^{2})}\right],\\ \mathrm{where}\;\mu_{f}-\mu_{g}= & y_{t_{i}}-H_{\alpha ,t_{i}}-H_{\beta ,t_{i}}m_{t_{i-1}}\;\mathrm{and}\;\sigma_{f}^{2}+\sigma_{g}^{2}=\sigma_{\nu }^{2}+{H_{\beta ,t_{i}}}^{2}c_{t_{i-1}}^{2}+\Xi_{t_{i}}. \end{array}$$
3 Create and store both $m_{t_{i}}$, and $C_{t_{i}}$,
  $$\begin{array}{cc} \mathrm{where}\;m_{t_{i}}= & H_{\alpha ,t_{i}}+H_{\beta ,t_{i}}m_{t_{i-1}}+\frac{{H_{\beta ,t_{i}}}^{2}c_{t_{i-1}}^{2}+\Xi_{t_{i}}}{{H_{\beta ,t_{i}}}^{2}c_{t_{i-1}}^{2}+\Xi_{t_{i}}+\sigma_{\nu }^{2}}\left[y_{t_{i}}-H_{\alpha ,t_{i}}-H_{\beta ,t_{i}}m_{t_{i-1}}\right]\\ \mathrm{and}\;c_{t_{i}}^{2}= & {H_{\beta ,t_{i}}}^{2}c_{t_{i-1}}^{2}+\Xi_{t_{i}}-\frac{{\left({H_{\beta ,t_{i}}}^{2}c_{t_{i-1}}^{2}+\Xi_{t_{i}}\right)}^{2}}{{H_{\beta ,t_{i}}}^{2}c_{t_{i-1}}^{2}+\Xi_{t_{i}}+\sigma_{\nu }^{2}}. \end{array}$$
4 Increment *i*, *i* = (*i* + 1) and repeat steps 2–3 till $\log\pi (y_{t_{N}}|y_{t_{1:(N-1)}})$ is evaluated.
5 Calculate the sum:
  $$\log\pi (y_{t_{1:N}})=\sum_{i=1}^{N}\log\pi (y_{t_{i}}|y_{t_{1:(i-1)}}).$$

## Appendix E Parameter posterior diagnostic plots

See Fig. E.1, Fig. E.2.

## Appendix F Computational speed statistics

See Table F.1
